# Clustered Volleys Stimulus Presentation for Multifocal Objective Perimetry

**DOI:** 10.1167/tvst.11.2.5

**Published:** 2022-02-03

**Authors:** Corinne F. Carle, Andrew C. James, Faran Sabeti, Maria Kolic, Rohan W. Essex, Chris Shean, Rhiannon Jeans, Aiasha Saikal, Alice Licinio, Ted Maddess

**Affiliations:** 1 John Curtin School of Medical Research, Australian National University, Canberra, Australia; 2 Optometry and Vision Science, University of Canberra, Canberra, Australia; 3 Canberra Hospital, Canberra, Australia

**Keywords:** multifocal, objective, perimetry

## Abstract

**Purpose:**

Multifocal pupillographic objective perimetry (mfPOP) is being developed as an alternative to subjective threshold perimetry for the management of visual and neurological disorders. Here, we evaluate, in normal subjects, differences in signal quality between the original mfPOP method of spatially sparse Continuous stimulus presentation and the new Clustered Volleys (CVs) method. We hypothesized that the CVs method would lead to increased signal-to-noise ratios (SNRs) over the original method due to the stabilization of gain within the pupillary system.

**Methods:**

Data were collected from six separate studies where otherwise-identical pairs of mfPOP tests using either the original Continuous stimulus presentation method or the new CVs method were undertaken; 440 6-minute tests from 96 normal subjects of varying ages were included. Per-region SNRs were compared between the two methods.

**Results:**

Mean SNRs for the CVs mfPOP variants were between 35% and 57% larger than the original Continuous mfPOP variants (*P* < 0.001 in five of six studies). Similarly, the goodness-of-fit measure (*r*^2^) demonstrated large and significant fold increases of between 2.3× and 3.4× over the original method (all *P* < 0.001). Significant improvements in SNRs were present in all of the 88 test regions (44/eye), ranging between 8.4% and 93.7%; mean SNRs were significantly larger in 98% of test subjects.

**Conclusions:**

The CVs mfPOP stimulus presentation method produced substantial increases in signal quality over the original method. This is likely due to the stabilization of pupillary gain during stimulus presentation.

**Translational Relevance:**

These improvements increase diagnostic accuracy and have enabled shorter, 80-second mfPOP tests to be developed.

## Introduction

Multifocal pupillographic objective perimetry (mfPOP) is an emerging technique for the diagnosis and management of disorders involving the visual system. Although pupillography and pupil perimetry using the presentation of isolated stimuli has been explored by many different groups,^1^^–^^4^ multifocal pupil perimetry, with its concurrent and repeated presentation of stimuli at many visual field locations, has been less widely investigated.^5^^–^^8^ The mfPOP method evolved from spatially sparse stimulus presentation methods originally developed for multifocal visual evoked potentials (mfVEPs).^9^ These sparse mfVEP methods have been demonstrated to greatly increase signal-to-noise ratios (SNRs) compared with traditional methods,^9^ in addition to improving diagnostic power.^10^ Spatially sparse mfVEP and mfPOP methods have been shown to produce similar diagnostic accuracy to each other in age-related macular degeneration (AMD)^11^ and multiple sclerosis.^10^^,^^12^ Both of these multifocal techniques use a regressive method to extract response estimates from the evoked responses and provide standard errors for those estimates, thus allowing per-region SNRs to be estimated in the form of *t*-statistics.

For mfPOP recording of pupillary responses, test durations of 4 or 6 minutes have been used, and both eyes are tested concurrently. Responses are reported relative to the subject's baseline pupil diameter. This normalization to a population average of 3.5 mm reduces the effect of the smaller pupil diameters^13^ and consequent smaller constrictions^14^ that occur in older subjects. Breaks in pupil recordings due to blinks and fixation losses are automatically compensated for, greatly reducing the need to repeat stimuli. Strong evidence has been provided that the transient onset stimuli used in mfPOP primarily drive the cortical pathway to the pupil.^11^^,^^15^^–^^17^ Promising findings have been reported using mfPOP in multiple sclerosis,^12^ concussion,^18^ migraine,^19^ glaucoma,^16^^,^^20^^,^^21^ AMD,^22^^–^^24^ and early-stage diabetic retinopathy.^8^^,^^25^

**Figure 1.**
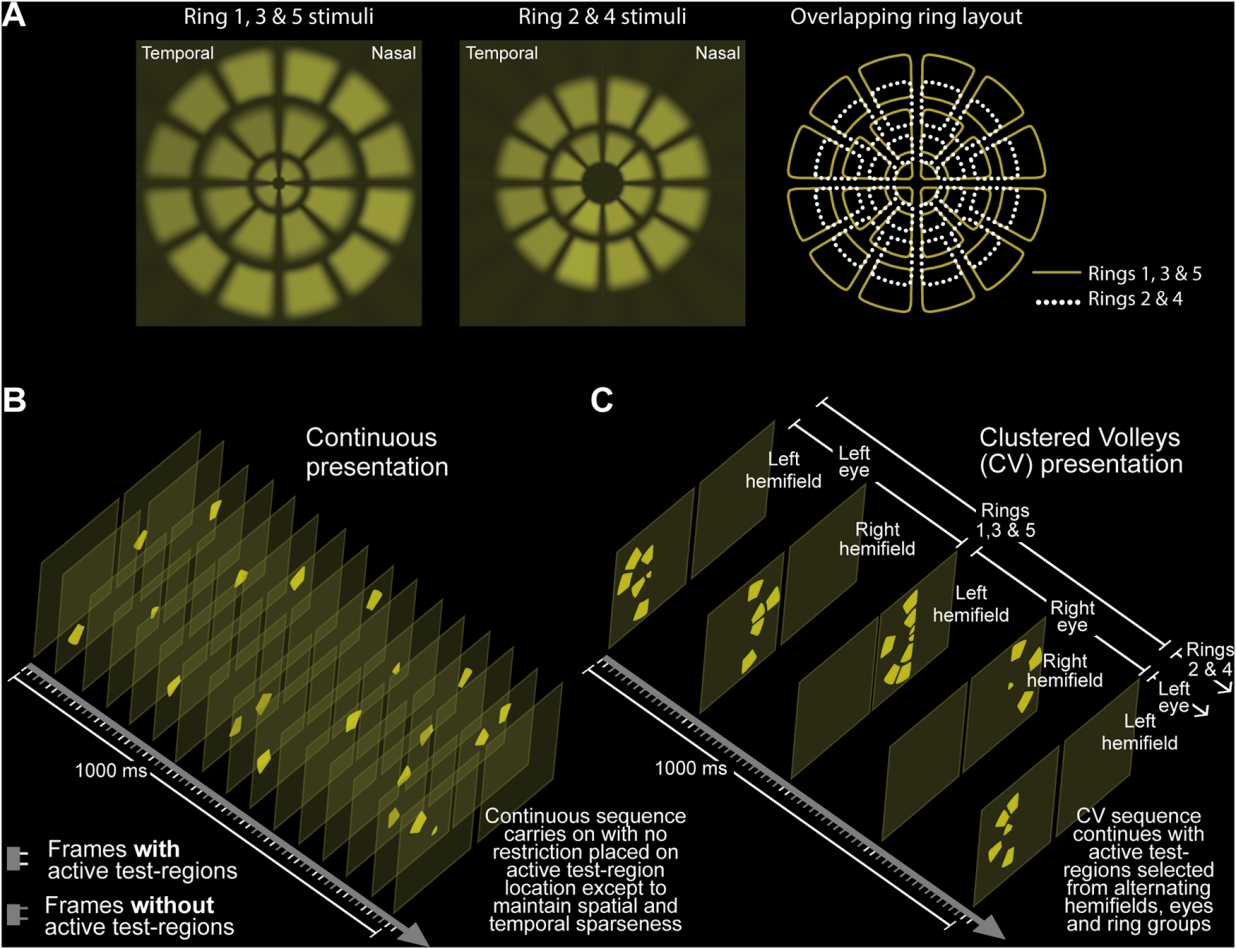
(A) MfPOP 44-region test region layouts. Test regions overlap slightly as shown at the *right* and extend to either a 60° or 30° visual field, depending on the stimulus variant (Table). The subtle variation in luminance between test regions in this layout is due to the use of luminance balancing. (B) One-second sample of stimulus frames for the Continuous presentation method. (C) One-second sample of stimulus frames for CVs presentation. Annotations (hemifields, eyes, rings) at right refer to the restrictions placed on stimulus location in this method. Each frame shown in (B) and (C) represents two frames of 16.67-ms duration to obtain the 33-ms duration stimuli. Intervening frames containing no active test regions are not shown in this diagram. The mean interval between stimulus presentations within each test region was 4 seconds in both presentation methods. (For further information, please refer to Supplementary Figs. S1–S5).

The mfPOP method has developed into a range of variants providing different utilities and generating steady improvements in SNRs. Original methods presented 24 concurrent stimuli to each eye,^7^^,^^26^ evolving to 40^21^ and then 44 somewhat overlapping stimuli,^27^ which ameliorate the effects of spatial undersampling^28^ and increase SNRs and diagnostic power.^22^^,^^23^ Macular variants have been developed for AMD,^23^^,^^24^ and photopic and scotopic versions have been compared,^26^ as well as many color^16^^,^^17^^,^^20^^,^^27^ and flicker variants.^24^^,^^26^^,^^29^ Newer mfPOP variants incorporate luminance balancing (Fig. 1A), in which brighter stimuli are presented in parts of the visual field that in normal subjects are intrinsically less sensitive, and dimmer stimuli are presented in regions that are more sensitive. Due to the effects of pupillary gain, which is governed by the overall visual input to the system,^30^ less intense stimulation of highly sensitive regions causes the amplitudes of less-sensitive regions to be boosted.^20^^,^^23^ This occurs because the concurrent presentation of stimuli results in a gain-modulated summed response in which individual response estimates are somewhat smaller than the product of the number of test regions stimulated and the response to an isolated stimulus.^21^^,^^30^^–^^33^

All of these early methods used so-called Continuous stimulus presentation, which evenly and sparsely samples the visual field across both space and time (Fig. 1B).^34^ A mfPOP model rationalizing contraction anisocoria^33^ and investigations of gain control,^30^ however, have highlighted the need to consider pupillary gain and its impact on the composite response. We surmised that reducing the frequency of changes in the gain state during the response time course, by presenting stimuli in a different manner, might improve SNRs and the accuracy of our response estimation. From that work has come a new Clustered Volleys (CVs) stimulus presentation method (Fig. 1C).^35^ A study of 40 patients with AMD and 23 control subjects (71.3 ± 5.1 years) demonstrated that the CVs method increased SNRs and diagnostic power compared with continuous presentation.^24^ The current study sought to more broadly quantify any change in SNRs produced by CVs by examining 440 mfPOP tests (880 fields) from 96 visually healthy subjects of widely varying ages. Both widefield and macular variants were examined.

## Methods

### Participants

We analyzed data from normal control subjects taken from six studies in which the Continuous and CVs presentation methods were compared head to head (Table). The first study, Norm1, was the initial proof of principle. Norm2 aimed to replicate that initial finding in a larger group of control participants. Norm3 and Norm4 compared pupillary luminance–response functions between the two methods for 60° (widefield) and 30° (macular) stimulus layouts, respectively. The remaining data comprise the responses of control subjects from two studies comparing the diagnostic utility of each method for glaucoma (PrelimG) and macular degeneration (PrelimA). Thus, all findings presented here pertain to subjects with no detectable visual disease. The age, sex, and number of participants in each study are presented in the Table.

**Table.** Study and Test-Subject Characteristics

Study	Purpose	Field Type	mfPOP Variants (Luminance), *n*	Repeats, *n*	Age (y), Mean ± SD	Subjects (Male), *n*
Norm1	Exploratory study	60°	2 (150)	2	45.8 ± 13.9	5 (3)
Norm2	Confirmatory study	60°	2 (150)	2	25.9 ± 12.0	23 (12)
Norm3	Luminance response in healthy subjects	60°	8 (38, 75, 150, 300)	1	37.7 ± 19.7	6 (3)
Norm4	Luminance response in healthy subjects	30°	8 (36, 72, 144, 288)	1	21.0 ± 1.0	18 (9)
PrelimG	Normative data for preliminary glaucoma study	60°	2 (150)	2	66.0 ± 8.6	24 (12)
PrelimA	Normative data for preliminary AMD study	30°	2 (288)	1	70.2 ± 4.9	20 (8)
Total subjects						96 (47)

Luminance values refer to the maximum stimulus luminance of a test protocol. The number of repeats (*n*) refers to the number of times subjects underwent the same variant in a study. In all cases, half of the variants utilized Continuous presentation and the other half CVs, each pair being otherwise identical as per the parameters listed here.

Exclusion criteria included best-corrected visual acuity of less than 6/12, spherical equivalent correction worse than ±9 diopters (D) or cylinder worse than ±2 D, previous eye surgery (excepting uncomplicated cataract surgery), and the presence of ocular disease or any other condition that could affect pupil responses (including diabetes). The status of each participant was confirmed using fundus photography, frequency-doubling technology (FDT) Matrix perimetry or Humphrey achromatic visual fields, and optical coherence tomography. Informed written consent was given by all participants according to Australian National University Human Research Ethics Committee approval 238/04. All research adhered to the tenets of the Declaration of Helsinki.

### Stimuli

Each study was comprised of either one or a series of matched pairs of otherwise identical Continuous and CVs mfPOP variants (Table). Therefore, the only difference between the variants in each pair was the temporal sequence of the stimulus presentation method; all other stimulus parameters (e.g., luminance, stimulus and test duration) were identical. Across the studies each Continuous/CVs pair differed from other pairs by the maximum luminance of the stimuli presented or by the extent of the visual field that was assessed. Stimuli were presented at optical infinity on a 10-cd/m^2^ yellow background comprised of a radial grating generated by smoothly varying the luminance contrast by ±10%. This subtle variation aids binocular fusion of the left and right eye stimulus arrays (Fig. 1A). All studies utilized yellow luminance-balanced stimuli in which the stimulus luminance differed between test regions in order to optimize SNRs. This method produces constriction amplitudes that are more uniform in normal subjects by compensating for the differing density of photoreceptors across the retina and corresponding visual field.^20^^,^^23^ Subjects completed either one or two complete sets of tests (repeats; see Table), during which they fixated a small red cross at the center of the field. The mfPOP variants were presented in randomized order using a prototype of the objectiveFIELD Analyzer (Konan Medical USA, Irvine, CA). Details of the recording setup, stimulus presentation, and response estimation are shown in Supplementary Figures S1 to S5.

Stimuli were arranged in overlapping dartboard layouts (Fig. 1A) and were presented in nine 40-second segments with short breaks in between (240 seconds total); the total testing time for each variant was around 8 minutes. Approximately 7920 stimuli were shown in each test (i.e., 90 in each of the 44 test regions per eye). Each stimulus was of 33-ms duration; the mean interval between stimulus presentations in each test region was 4 seconds (Figs. 1B, 1C). Note that, although the presentation sequence differed between the two methods, the interval (i.e., temporal sparseness) and total number of stimuli shown were essentially the same in both.

### Clustered Volleys Stimulus Presentation

**Figure 2.**
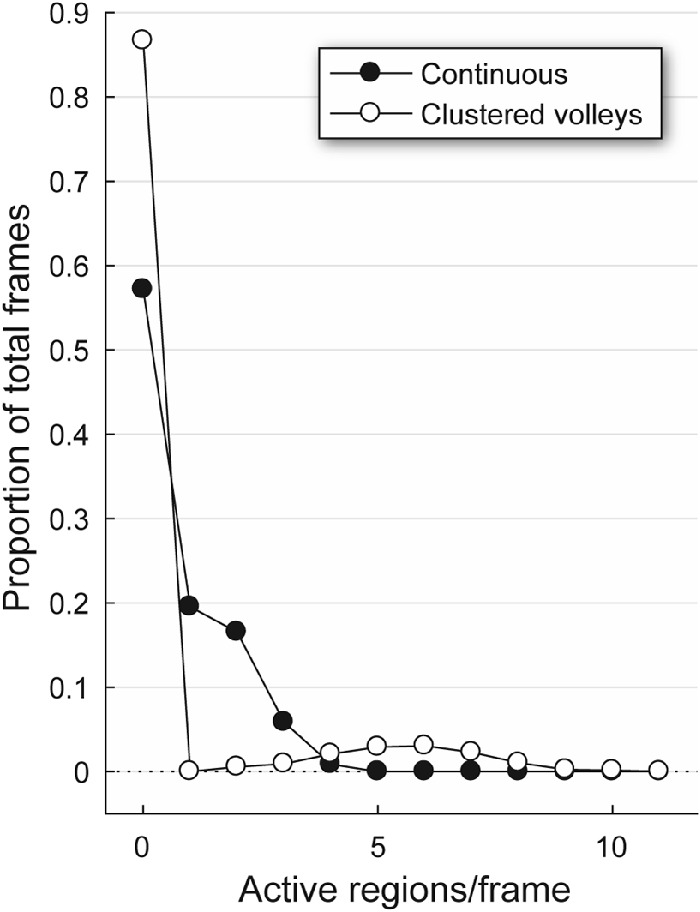
Proportion of frames in each test with a given number of active stimulus test regions. Note that the maximum number of concurrently presented regions is four for the Continuous method but can be as high as 11 using the CVs method (refer to Supplementary Figs. S2 and S3).

In order to reduce the frequency of changes in summed visual signal during the response time course and therefore move to stabilize the gain of the system, restrictions were placed upon when and where each of the CVs stimuli could appear. This involved presenting them in groups; that is, volleys appeared every 0.25 seconds rather than in a continuous stream. We achieved this by nesting sets of potential locations, first by ring group, then by eye, and finally by hemifield (Fig. 1C). Test regions from each of these subsets had a 50% probability of being active within a given stimulus volley. As with the original Continuous presentation method, the stimulus sequence within each of the 88 test regions was identical, but the start point in this sequence was different for each region; in a manner similar to that of an m-sequence, the pattern of presentations in each region was subject to a different rotational shift.^36^ This ensured the statistical independence of the presented stimuli and allowed for estimation of responses using our regressive method. With either 10 (stimulus rings 2 and 4) or 12 (rings 1, 3, and 5) potential test region locations within each volley, the number of stimuli appearing at the same time was higher using the CVs method (Fig. 2) and stimuli therefore were less spatially sparse. The synchronized onset of stimuli within each volley, however, was designed to reduce the constant changes in the gain state of the pupillary system that occur with Continuous presentation (Fig. 1B, Fig. 2), thus producing more robust responses. In addition, based on evidence of differing gain states dependent on origin in temporal or nasal retina,^33^ restricting the stimuli within each volley to single eyes and hemifields was surmised to further reduce unwanted noise.

### Data Analysis

The primary response variables investigated were the SNRs, defined as the ratio of fitted per-region response amplitudes on their standard error, for each subject and test (i.e., the *t*-statistics for direct and consensual responses in each test region). The goodness-of-fit measure (*r*^2^) was estimated as the variance explained by the regression model used to estimate responses divided by the total variance in the response. The regression analysis for each test incorporated left or right pupil responses to stimulation in all 88 test regions; thus, two *r*^2^ values were produced for each test on each subject. Linear regression was also used to investigate the differences in SNRs achieved between the Continuous and CVs methods. Inputs to the linear models comprised the means across pupils, eyes, repeats, and, where applicable, regions, thus producing extremely conservative significance estimates, as the models assume complete correlation between these response components. Multiple comparisons were compensated for by adjusting significance levels using Bonferroni correction. No factors were fitted for variables such as age or sex, so the model outputs for these comparisons are effectively *t*-tests.

## Results

**Figure 3.**
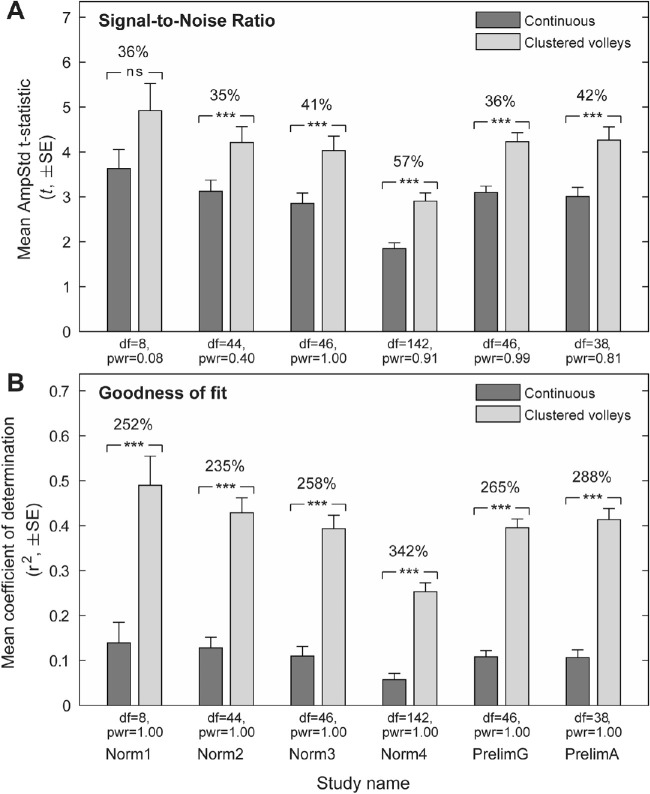
Overview of the difference in quality parameters between the Continuous and CVs presentation techniques. (A) Mean signal-to-noise ratios and (B) coefficients of determination (*r*^2^) are shown for pairs or sets of otherwise identical Continuous and CVs variants from each study. *Square brackets* indicate paired *t*-test; ***P < 0.001. Values above brackets represent the percent increase in CVs parameters relative to Continuous parameters. Degrees of freedom (df) and post hoc statistical power (pwr, α = 0.004) are shown for each comparison.

The mean SNRs for each study were substantially larger for all CVs variants than the original Continuous method. These differences were significant in all but the smallest study, Norm1, and represented mean increases of between 35% and 57% over the old method (Fig. 3A). The goodness-of-fit measure (*r*^2^), representing the proportion of variance in individual subject's pupil records that could be accounted for by the stimuli, showed even greater improvements than the SNRs. These represented highly significant fold increases in mean *r*^2^ values of between 2.3× and 3.4× (Fig. 3B).

**Figure 4.**
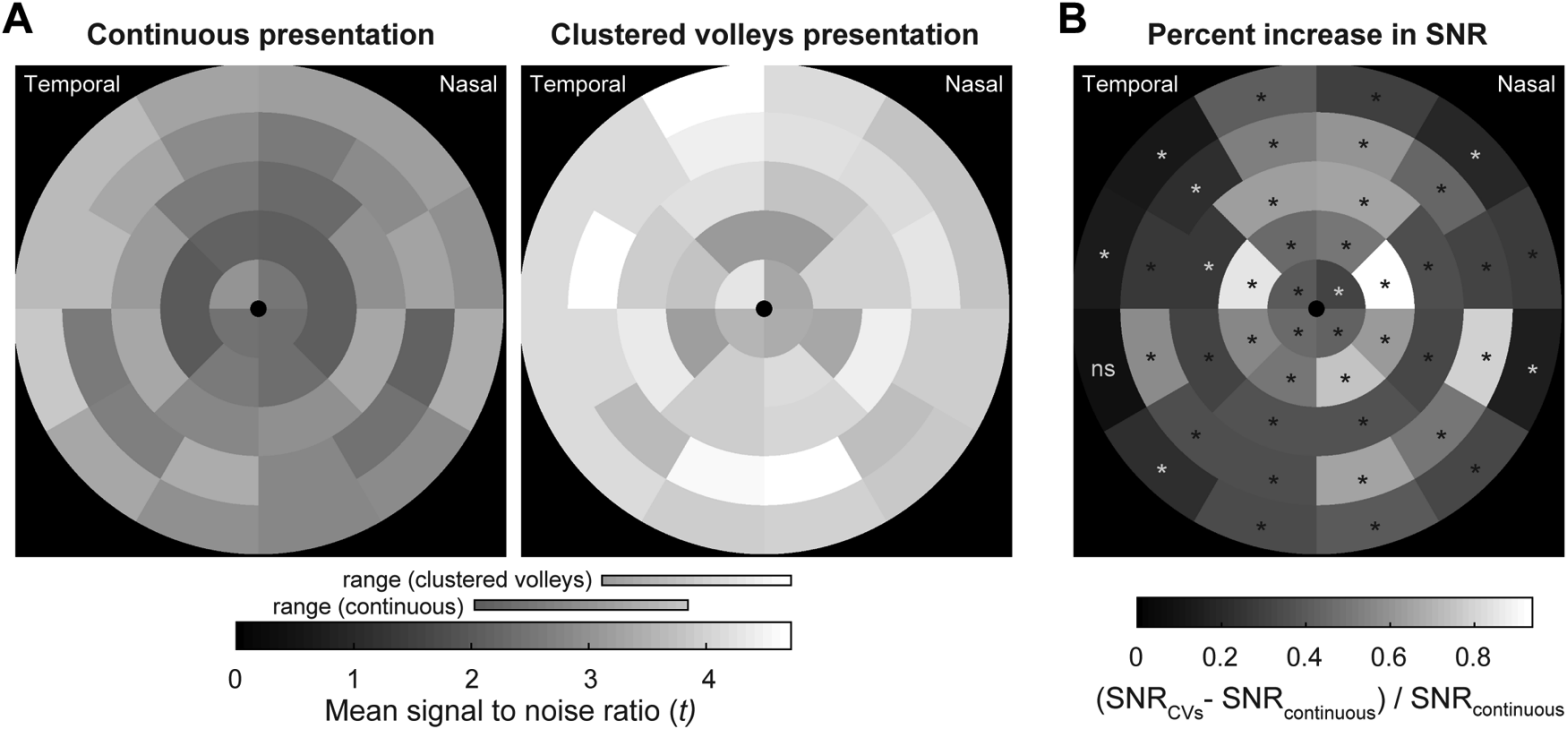
Topographic variation in SNRs and percent increases in SNRs between Continuous and CVs presentation techniques. (A) Mean SNRs (*t*-statistics) across all subjects and test protocols for each test region (right eye regions are shown as left-equivalent, with the temporal visual field at the left). (B) The proportional increase in regionwise SNRs in CVs presentation. Significant differences are marked with an *asterisk* (*P* < 0.0011, Bonferroni-corrected; ns = non-significant). The background color (*black*) in each panel represents a value of zero.

**Figure 5.**
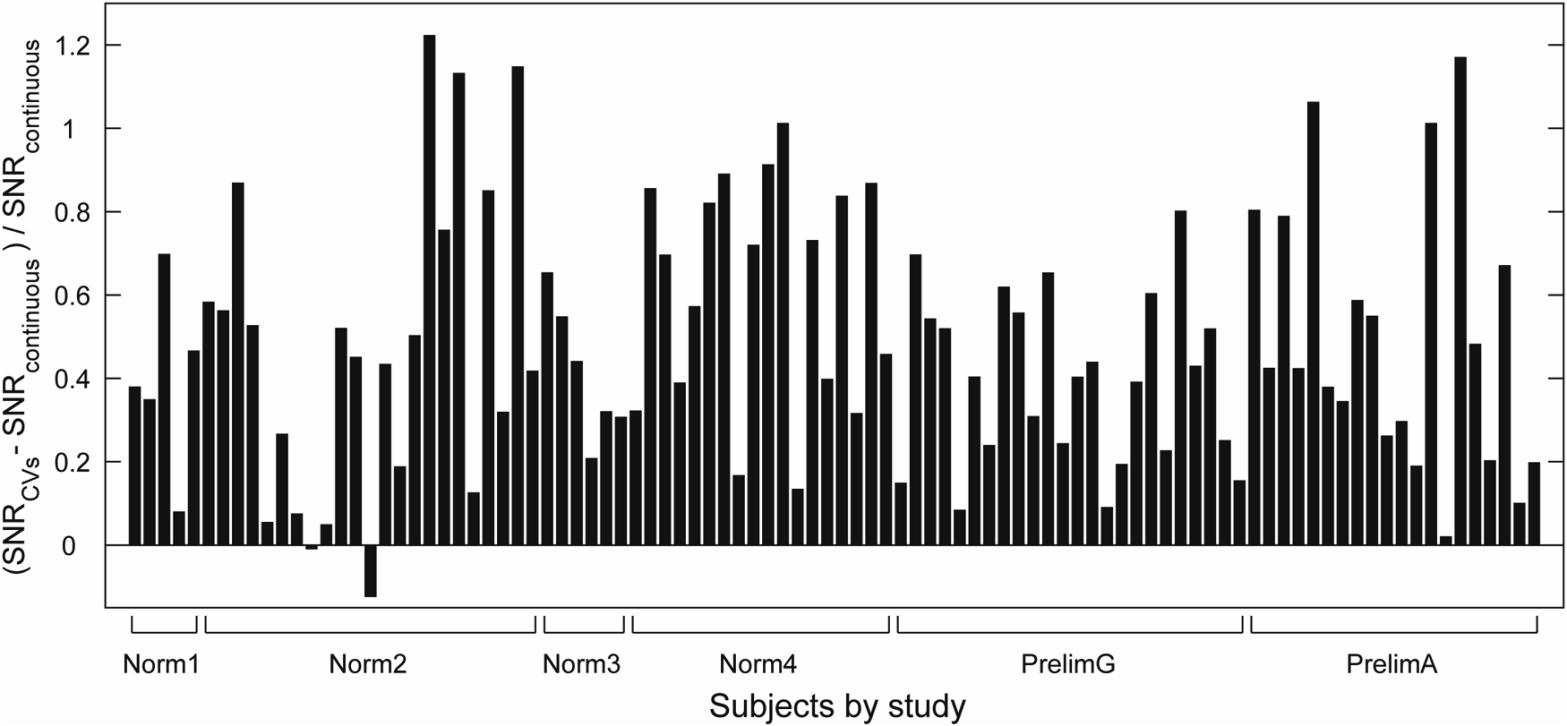
Percent increase in mean SNRs (*t*-statistics) across all test regions and test protocols for each subject, grouped by study. Ninety-eight percent of subjects demonstrated increased mean SNRs using the CVs method relative to Continuous presentation. Some variability is due to a range of stimulus intensities being used within some studies (Table).

These improvements are reflected across the entire stimulus array. Means of SNRs for individual test regions of CVs variants were more uniform across the visual field than those produced using the Continuous method, and their range, although overlapping, was substantially higher (Fig. 4A). Mean CVs SNRs were larger in every stimulus test region (Fig. 4B), this being more pronounced in test regions that produced lower SNRs using the original method. Percent increases ranged from 8.4% (*P* = 0.038) in the temporal field to 93.7% (*P* < 0.00005) more centrally, with a median SNR increase of 39.0% across all regions. A similar pattern can be seen across individual subjects, with 98% producing larger mean SNRs with the CVs method (Fig. 5). Only one individual produced significantly lower mean SNRs from the CVs method than from the Continuous method. The median improvement across all subjects was 43.7% (range, −12% to 122.4%)

## Discussion

Maximizing the clinically useful information that is contained within a test output is essential to the reliability and accuracy of any diagnostic tool. Regardless of the type of test, this so-called signal will always be accompanied by meaningless information, or noise, resulting from unidentifiable sources of variance such as measurement error, estimation error, and, of course, physiology. The ratio between these two factors, the SNR, can sometimes be maximized by increasing the stimulus intensity and thus the magnitude of the response, but this comes with its own risks. Often the noise increases along with the signal, and eliciting a large response that sits within the upper saturated range of a sigmoidal stimulus–response curve can render small changes in function invisible. Similarly, a high proportion of noise in measurements necessitates larger sensitivity losses to achieve robust and statistically significant observations. Thus, a careful balancing act is required between maximizing the SNR (and therefore confidence in your measurements) and conserving diagnostic sensitivity.

The mfPOP method holds a unique place in pupil perimetry due to its ability to obtain measures of SNR, this arising from the regressive method used to estimate responses.^9^ Other forms of multifocal pupil perimetry, such as those explored by Wilhelm et al.^6^ and Tan et al.,^5^ were somewhat limited by the use of cross-correlation to estimate responses as per Sutter's original m-sequence method.^36^ Cross-correlation of responses from simultaneous time series can be somewhat confounded due to autocorrelation in the stimulus sequences. The issues and limitations arising from this were modeled nicely by Dean and Dunsmuir.^37^ Theoretically, SNRs can be obtained from focal presentation of stimuli^2^^–^^4^; however, the time taken to obtain numerous measurements in each visual field location limits the number of regions that can be tested or otherwise results in unreasonably long test durations.

Several factors influence the pattern of pupil constrictions in mfPOP. First, of course, is the integrity of the visual system; intact, healthy populations of cells produce larger constrictions than do diminished, diseased populations.^21^^,^^38^ Similarly, higher luminance and longer duration stimuli also produce larger amplitudes within a given range.^27^ mfPOP constriction amplitudes, curiously, are also modulated by the spatial and temporal density of stimuli.^30^^,^^39^ This manifests as a divisive gain mechanism and is evidenced by the effect of the summed visual signal on the amplitude of individual mfPOP pupil constrictions; we have previously reported on the nature of this summation in the context of contraction anisocoria^33^ and gain control in the pupillary system.^30^ Similar effects have been observed using multifocal visual evoked potentials.^40^^–^^42^

The original method of spatially sparse mfPOP utilized Continuous stimulus presentation, which involves displaying stimuli in a pseudorandom sequence with different temporal offsets for each of the 88 visual field test regions (Fig. 1B). However, the time course of individual pupil constrictions to 33-ms mfPOP stimuli is around 400 ms,^16^ and as many as six stimulus frames, each containing different numbers of stimuli (Fig. 2), can be presented within the window of each constriction. Therefore, using this older method, as many as six composite responses may overlap with each other, each at different stages of their time course. Although the Continuous method produces stimulus streams that are statistically independent and has been shown to have considerable diagnostic utility, this overlap and the constant fluctuations in the gain state of the pupillary system that result are a potential source of noise in the mfPOP signal. We therefore reasoned that the quality of mfPOP responses could be improved by minimizing these changes and, having developed the CVs presentation method to address this, have demonstrated here substantial improvements in the quality of the fitted responses (Fig. 3B) and their SNRs (Figs. 3A, 4, 5).

**Figure 6.**
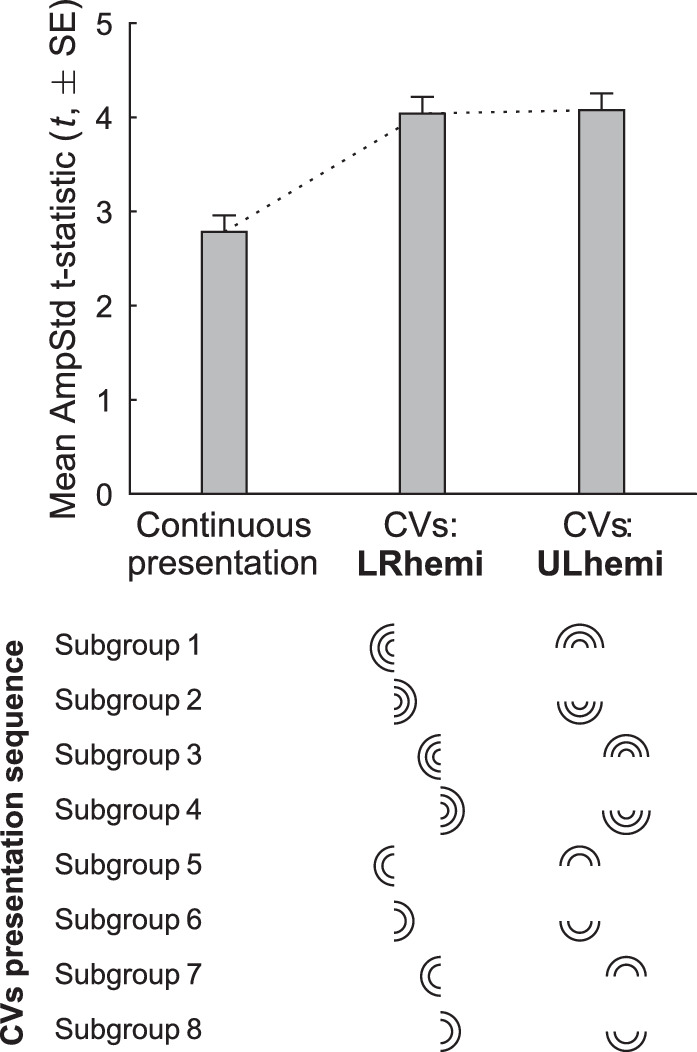
Mean SNRs (*t*-statistics) of standardized constriction amplitudes (AmpStd) for the Continuous presentation method and two CVs variants (23 subjects). Shown below the *bar plot* is the presentation sequence for the two CVs variants, the first (LRhemi) utilizing stimuli from alternating left and right hemifields, the second (ULhemi) utilizing alternating upper and lower hemifields. Sets of three arcs represent the stimuli in rings 1, 3, and 5 for the appropriate hemifield and eye. Sets of two arcs represent stimuli in rings 2 and 4 (refer to Figs. 1A and 1C for nesting of hemifields, eyes, and rings during the CVs presentation).

The second factor that we surmised may have contributed to variability is the differing effect on pupillary gain of signal originating in nasal or temporal retina. We have previously proposed a model rationalizing contraction anisocoria based on observed differences between relative proportions of direct and consensual responses dependent on hemiretina of origin.^33^ We have since undertaken an investigation of this using CVs stimuli that alternate between either left and right hemifields, as used in this paper, or between upper and lower hemifields. Because the gain control proposed in our anisocoria model varied depending on whether or not the retinal projection decussated at the optic chiasm, these two conditions would produce slightly different results if the observed improvements in SNR were due to decussation-specific differences in midbrain summation of signal. Preliminary results are shown in Figure 6, where there appears to be little, if any, difference between the two CVs variants; both show similar improvements from the older Continuous method. It would seem, therefore, that the improvements we have observed in the CVs method are mostly, if not solely, due to the partial stabilization of gain state by presenting stimuli in volleys rather than in a continuous stream.

Other factors, of course, have the potential to contribute to variability in the pupil response. These include the slow oscillations that characterize both hippus and accommodative fluctuations.^43^^,^^44^ In developing the mfPOP method, we have taken steps to minimize these phenomena; stimuli are presented at optical infinity and blurred to render small changes in depth of field or defocus negligible. Additionally, testing occurs in short segments that lessen fatigue. Attentional focus also has been observed to modulate pupil responses, although this effect is less evident using yellow stimuli, which are standard in mfPOP.^45^ Although the pupillary response is often simplistically conceptualized as a basic reflex loop, in addition to direct retinal input from intrinsically photosensitive retinal ganglion cells the pretectal component of the pupillary pathway receives inputs from striate, extrastriate, and frontal cortex, as well as thalamic and other midbrain nuclei.^46^ In line with those anatomical findings, we have reported that per-region hypersensitivities observed in mfPOP fields, associated with earlier stage retinal disease, are also observed in head-to-head mfVEP fields when electrodes recording the extrastriate, but not striate, cortex are used.^11^

In conclusion, the data presented here from 96 subjects clearly indicate that the CVs method of stimulus presentation has substantial advantages over the earlier Continuous method. The improvements have led to the establishment of two CVs protocols as standard mfPOP tests: P129, a 60°, 150-cd/m^2^ variant; and P131, a 30°, 288-cd/m^2^ variant. Using these we have undertaken a number of projects regarding the diagnosis and management of visual and neurological disorders. In addition to these established tests, we have utilized the increased signal quality to develop much shorter 80-second tests; four variants are currently under investigation and show much promise.^47^^–^^49^

## Supplementary Material

Supplement 1

Supplement 2

Supplement 3

Supplement 4

Supplement 5
