# Supplementary material for: Clustered Volleys Stimulus Presentation for Multifocal Objective Perimetry
Source: Transl Vis Sci Technol. 2022 Feb 3;11(2):5. doi: 10.1167/tvst.11.2.5 (PMC8819283; doi:10.1167/tvst.11.2.5)
Supplement: Supplement 1 [file tvst-11-2-5_s001.pdf]

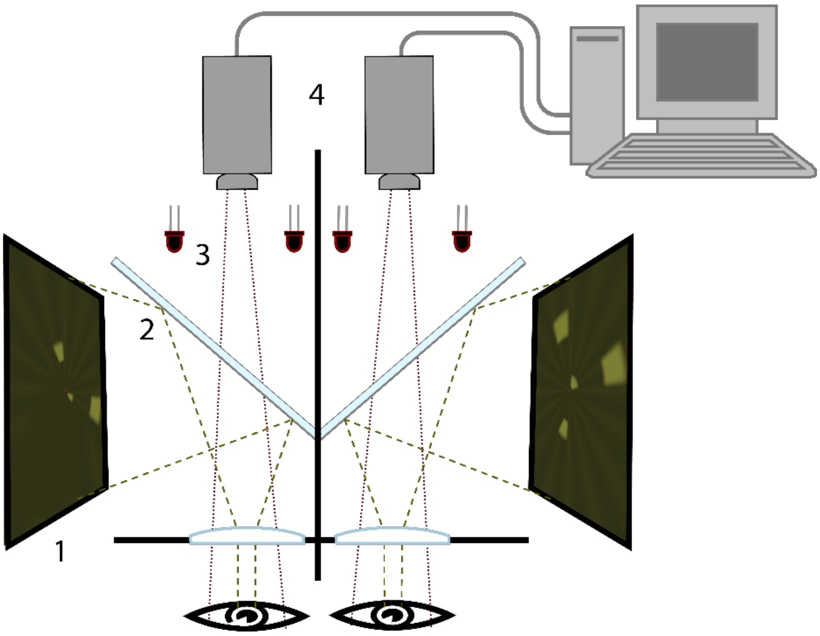

Supplementary Figure S1: Recording of mfPOP responses using a prototype OFA. Multifocal stimuli are presented dichoptically on liquid crystal displays (1) and reflected using cold-dichroic mirrors (2). Near-infrared LEDs (3) are used to illuminate the subject's eyes and the pupillary response is monitored using two infrared video cameras.
