# Supplementary material for: Clustered Volleys Stimulus Presentation for Multifocal Objective Perimetry
Source: Transl Vis Sci Technol. 2022 Feb 3;11(2):5. doi: 10.1167/tvst.11.2.5 (PMC8819283; doi:10.1167/tvst.11.2.5)
Supplement: Supplement 2 [file tvst-11-2-5_s002.pdf]

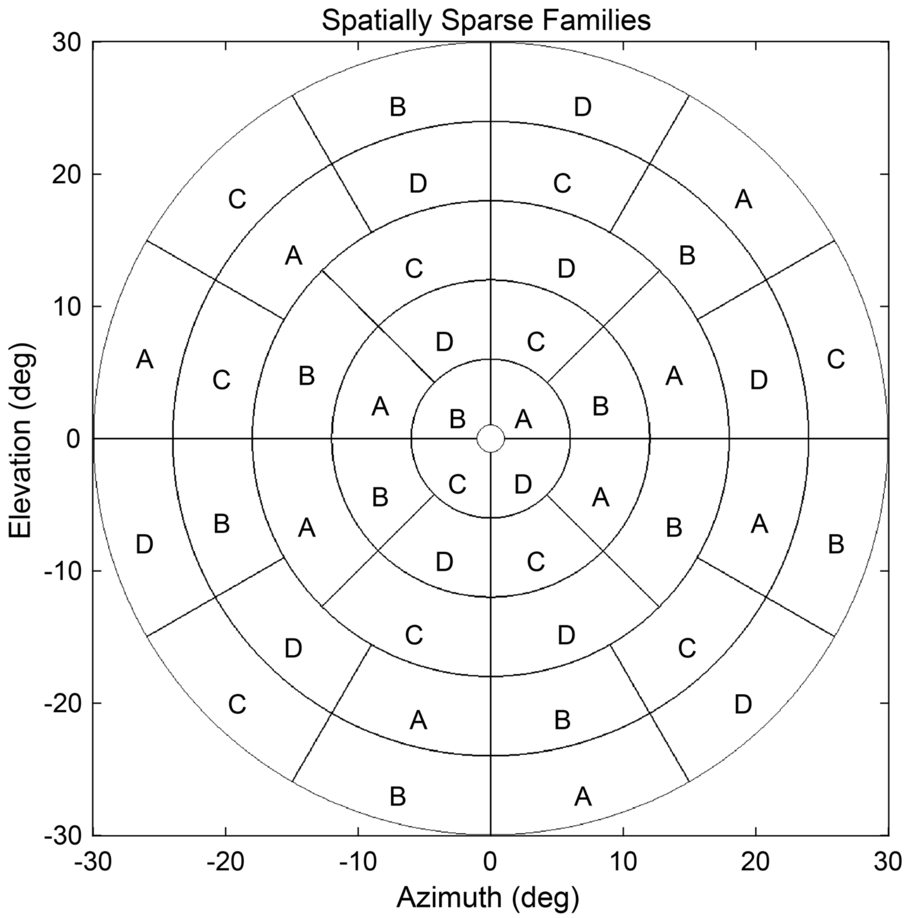

Supplementary Figure S2: Test-region selection using the Continuous method of sparse stimulus presentation. The 88 test-regions across both eyes are assigned to one of four families, such as A to D above. The layout of these ensures that no two members of the same family share an edge. Restricting concurrent stimulus presentations to a maximum of one from each group prevents laterally-adjacent test-regions from being active at the same time. Note that this restriction means that a maximum of four out of the 88 stimuli used in this experiment can potentially appear at a given time (Fig. 2). The stimuli are thus said to be spatially sparse, reducing the effects of cortical lateral suppression.

The same pseudorandom stimulus sequence is used to determine the presentation of active stimuli in each test-region, however, these sequences are subject to one of a number of different temporal offsets i.e. the same sequence is used to determine the potential appearance of stimuli in each of the 88 test-regions in this experiment, but this sequence is cyclically shifted to distribute the start-points relatively evenly across the duration of the test. An additional offset one of the following 30 values: 0s, 33.3 ms, 66.7 ms …. 2000 ms is also applied. The test-regions assigned to these latter offset values are selected to alternate between left and right eyes, with no more than one test-region from each of the four families (A-D) sharing the same offset. Potentially active regions then have a 50% probability of displaying a stimulus. Thus, in the current study, active stimulus presentations occurred in each test-region once every 4 s on average. Further details of the method are given in US patent US 8,585,223, which is freely available: Maddess, T & James, AC (2003). Assessment of neural function. US8583223, EP1694206, JP4764349, CA2547017.
