# Supplementary material for: Clustered Volleys Stimulus Presentation for Multifocal Objective Perimetry
Source: Transl Vis Sci Technol. 2022 Feb 3;11(2):5. doi: 10.1167/tvst.11.2.5 (PMC8819283; doi:10.1167/tvst.11.2.5)
Supplement: Supplement 3 [file tvst-11-2-5_s003.pdf]

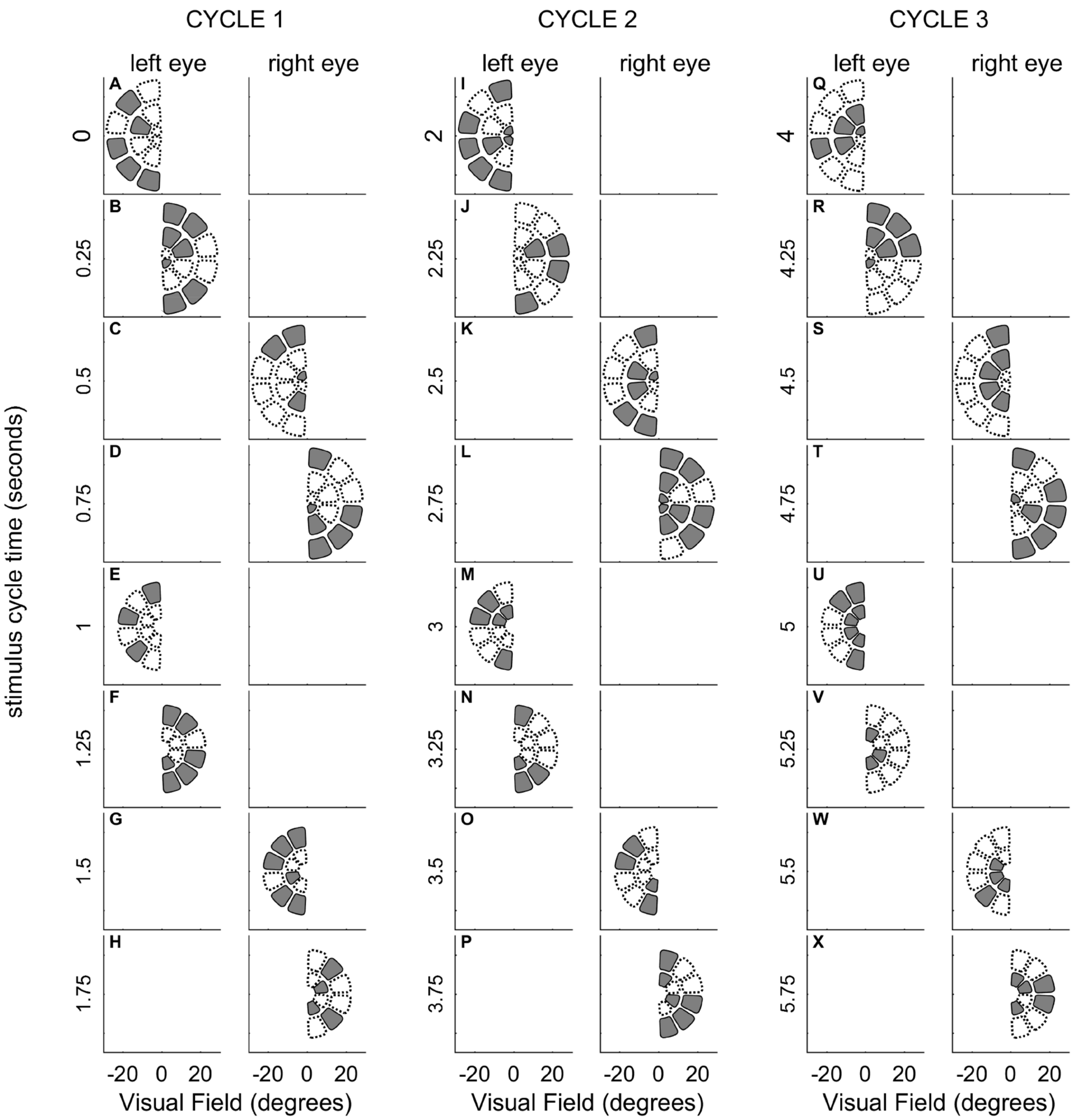

Supplementary Figure S3: Test-region selection using the Clustered Volleys method of stimulus presentation. Shown here is a six second extract from a Clustered Volleys sequence showing the nesting of test-region families within ring groups (1, 3 & 5 or 2 & 4), eyes (left or right), and hemifields (left or right). In this figure active regions are shown in grey, and inactive regions i.e. those not displaying a stimulus, as a dotted border. Volleys of stimuli appear every 0.25 s, each cycle takes 2 seconds in total (3 cycles are shown here). As with the Continuous method (Fig. S2), the stimulus sequence is the same for all test-regions, with temporal offsets applied to the sequence in each region to distribute the start-points across the test duration: in this case however, the additional offsets occur at 0 ms, 250 ms, 500 ms …. 2000 ms. This results in much higher temporal sparseness, but lower spatial sparseness due to the larger number of test-regions that can potentially be active, up to 11 for rings 1, 3 and 5 families, and up to 9 for rings 2 and 4 families (Figure 2). Again, test-regions within each volley have a 50% probability of being active i.e. displaying a stimulus, thus each test-region will have a stimulus presented every 4 seconds on average. Details of the Clustered Volleys method are given in US patent US9848771: Maddess T, Carle CF & James AC (2012). Clustered Volley Method. AU2013350326, CA2888423, CN105208917, EP2922461, JP2016501574, US9848771. Assigned to Konan Medical USA Inc.
