# Supplementary material for: Clustered Volleys Stimulus Presentation for Multifocal Objective Perimetry
Source: Transl Vis Sci Technol. 2022 Feb 3;11(2):5. doi: 10.1167/tvst.11.2.5 (PMC8819283; doi:10.1167/tvst.11.2.5)
Supplement: Supplement 4 [file tvst-11-2-5_s004.pdf]

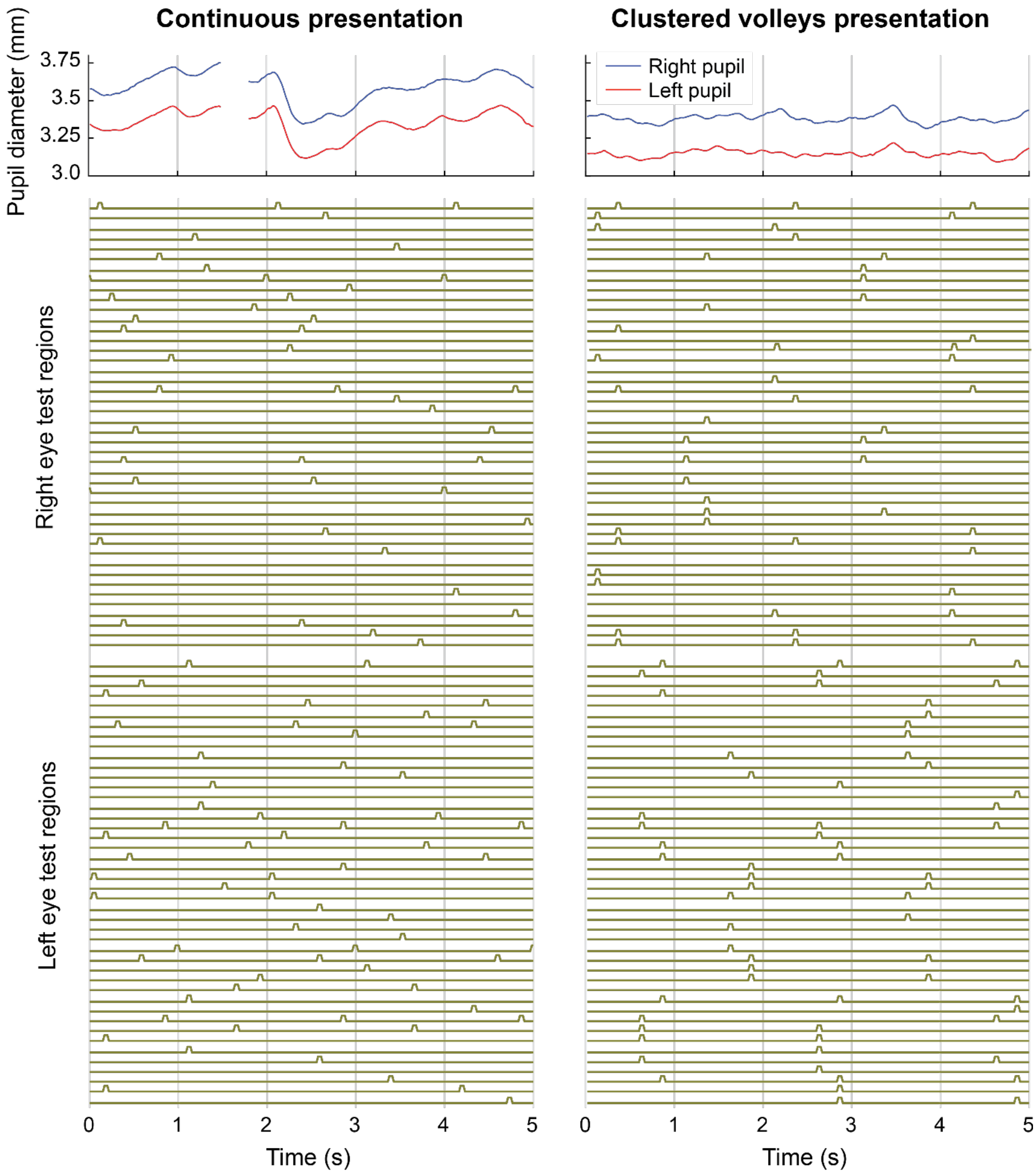

Supplementary Figure S4: The top panel shows a 5 s extract from the 360 s raw pupil record of a single subject to Continuous and Clustered Volleys mfPOP tests. Note that recordings are made simultaneously from both left (red trace) and right (blue trace) pupils. The gap in the traces in the Continuous example reflects missing data due to a blink. Blinks, unless excessive, have minimal impact on the results due to the regressive method used to estimate responses. The stimulus sequences for the corresponding time period for the 44 right eye and 44 left eye test-regions is shown in the lower panels. Upward deflections in each yellow trace represent the occurrence of stimulus presentations in that particular visual field test-region. The differences in spatial sparseness and structure between the two methods can be clearly seen here, even though stimuli in both methods are presented every 4 s on average in each test-region. The raw continuous pupil record is regressed onto this set of sequences to allow estimation of what are effectively mean responses to the 90 stimulus presentations in each test-region in these 360 s tests.
