# Supplementary material for: Clustered Volleys Stimulus Presentation for Multifocal Objective Perimetry
Source: Transl Vis Sci Technol. 2022 Feb 3;11(2):5. doi: 10.1167/tvst.11.2.5 (PMC8819283; doi:10.1167/tvst.11.2.5)
Supplement: Supplement 5 [file tvst-11-2-5_s005.pdf]

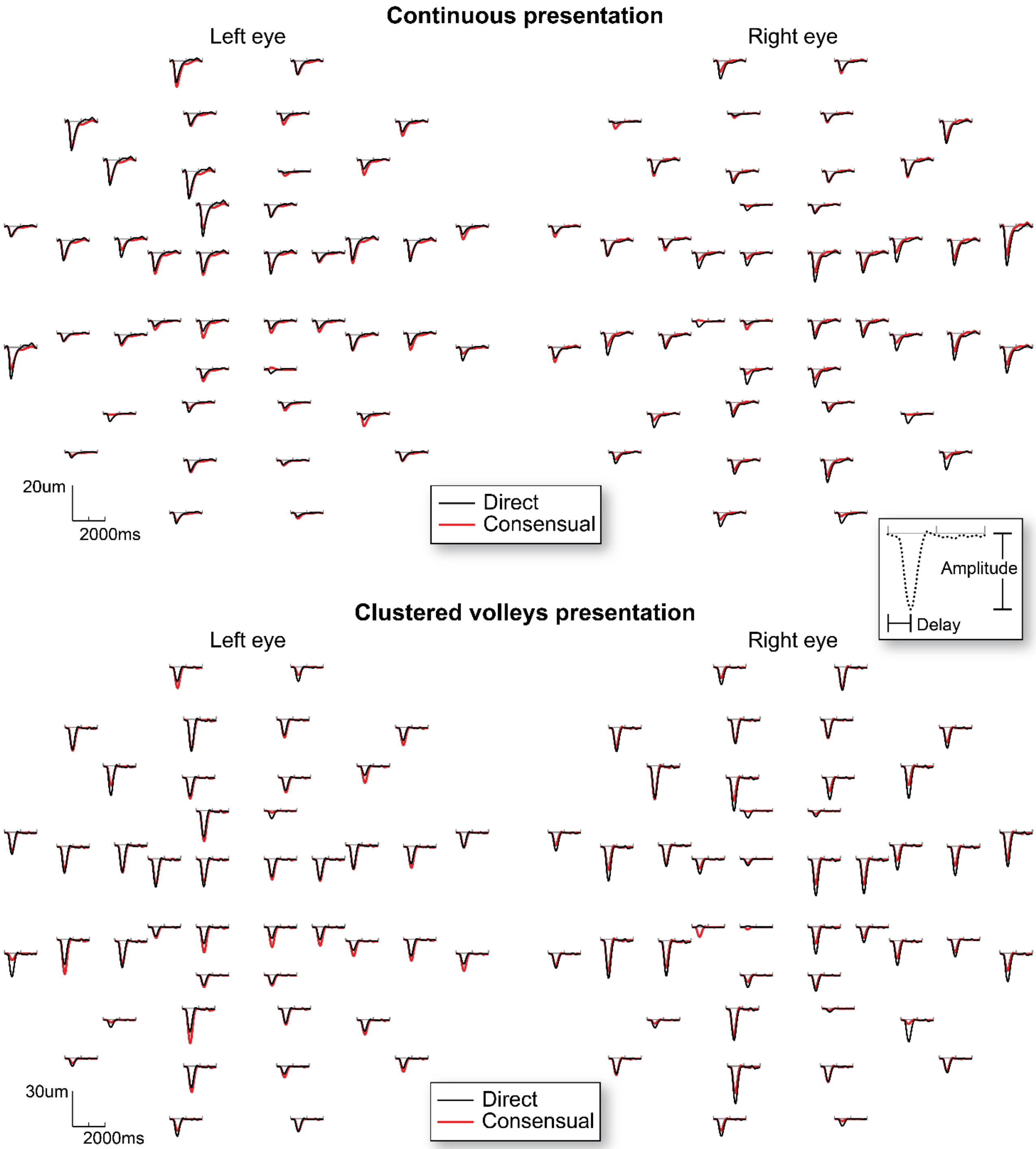

Supplementary Figure S5: Estimated responses for the same subject as in Fig S2 for the completed Continuous and CVs tests (5 s extract only shown in Figure S2; these estimates were made using the full 360 s record). Both Direct and Consensual responses are shown in this plot. Constriction amplitudes are calculated as the maximum deflection from baseline pupil diameter. The larger amplitude of responses using the Clustered Volleys method can be seen in this subject's responses (refer scale for each visual-field array). These response estimates can be used to generate maps of relative pupillary visual-field sensitivity or delay when compared against normative data.
